# Serum and brain metabolomic study reveals the protective effects of Bai-Mi-Decoction on rats with ischemic stroke

**DOI:** 10.3389/fphar.2022.1005301

**Published:** 2022-11-24

**Authors:** Lingling Yang, Xiaojuan Su, Fangfang Lu, Rong Zong, Shuqin Ding, Jing Liu, Gidion Wilson, Liuyan Li, Youyue Yang, Weibiao Wang, Xiaoying Wang, Jianyu Chen, Xueqin Ma

**Affiliations:** ^1^ Department of Pharmaceutical Analysis, Key Laboratory of Hui Ethnic Medicine Modernization, Ministry of Education, School of Pharmacy, Ningxia Medical University, Yinchuan, China; ^2^ Fujian University of Traditional Chinese Medicine, Fuzhou, China

**Keywords:** Bai-Mi-Decoction, ischemic stroke, pharmacodynamics, metabolomics, UHPLC-QTOF-MS/MS

## Abstract

Bai-Mi-Decoction (BMD), which is composed of *Eugenia caryophyllata*, *Myristica fragrans*, *Moschus berezovskii*, and *Crocus sativu*, is a characteristic TCM multi-herb formula for brain disease. However, the mechanism of protective effects of BMD on ischemic stroke (IS) still has not been clarified. Our study is designed to elucidate the protective effects and underlying mechanisms of BMD on IS by employing pharmacodynamic and serum and brain metabolomic methods. In this experiment, 90 adult male Sprague–Dawley rats were randomly divided into the sham operation group (SHAM, vehicle), middle cerebral artery occlusion–reperfusion injury model group (MCAO/R, vehicle), positive control group (NMDP, 36 mg/kg/day nimodipine), and low (BMDL, 0.805 g/kg/day), moderate (BMDM, 1.61 g/kg/day), and high (BMDH, 3.22 g/kg/day) dosage of BMD prophylactic administration groups. The drugs were dissolved in 0.5% CMC-Na and orally administered to rats with equal volumes (100 g/ml body weight) once a day for 14 consecutive days. Neurological deficit score, cerebral infarct volume, change in body weight, and serum NO, SOD, MDA, GSH, and GSSG levels were determined. Pathological abnormalities using hematoxylin and eosin staining and the expression of VEGF, caspase-3, and NF-κB were analyzed. Furthermore, serum and brain metabolic profiles were explored to reveal the underlying mechanism using UHPLC-QTOF-MS/MS technology. BMD exhibited significant neuroprotective effects on MCAO/R rats. As compared to the MCAO/R model group, it could reduce the neurological deficit score and cerebral infarct volume, increase body weight, enhance GSH, SOD, and GSSG activities, and decrease NO and MDA contents of MCAO/R rats. Meanwhile, BMD could ameliorate pathological abnormalities of MCAO/R rats through reducing neuronal loss, vacuolated spaces, shrunken neurons, and destructed neuron structure, as well as regulating the expression of VEGF, caspase-3, and NF-κB. UHPLC-QTOF-MS/MS-based serum and brain metabolomics analysis found a total of 53 differential metabolites between MCAO/R and SHAM groups, of which 30 were significantly regulated by BMD intervention, and further metabolic pathway analysis implied that the protective effects were mainly associated with amino acid and glycerophospholipid metabolisms. Our pharmacodynamic and metabolomic results revealed the neuroprotective effects of BMD on MCAO/R rats, and the underlying mechanisms were probably related to amino acid and glycerophospholipid metabolisms.

## 1 Introduction

Stroke is the second largest main cause of mortality and long-term disability worldwide, and it has developed as a major contributor for the overall global burden of healthy systems (Qureshi et al., 2016). Particularly in China, an estimated one million people died from stroke each year (Gairolla et al., 2017). The proportion of ischemic stroke (IS) accounts for approximately 85% of all stroke cases worldwide (Strong et al., 2007), which occurs when hypoxia is induced by the lack of blood flow in the middle cerebral artery of the brain (Cheon et al., 2018). Although the pathological process of IS was complex and still not clear, it was proved to be associated with energy depletion, inflammation, neuronal apoptosis, ionic imbalance, blood–brain barrier disruption, angiogenesis, oxidative stress, and neurological injury (Dirnagl et al., 1999; Cheon et al., 2018). Currently, thrombolytic therapy and antithrombic and antiplatelet medication were clinically used for treating IS (Fjetland et al., 2015; Chen et al., 2019a). Unsatisfactorily, the clinical application of these drugs has some limitations due to the narrow therapeutic time window and various kinds of adverse effects. Currently, with the increased acceptance of combination therapies for the treatment of complicated diseases, the development of traditional Chinese medicine (TCM) with characteristics of multi-components and multi-mechanisms have become an emerging trend.

Bai-Mi-Decoction (BMD) is a typical TCM multi-herb formula for brain diseases officially recorded in the Huihui Medical Formulary. It is composed of four herbs: *Eugenia caryophyllata* Thunb (EC), *Myristica fragrans* Houtt (MF), *Moschus berezovskii* Flerov (MB), and *Crocus sativus* L (CS). It had been used to cure paralysis, improve facial paralysis, and relieve physical weakness in clinical practice in the ancient time. The modern clinical practice revealed that BMD could improve the neurological function and daily living ability of patients with IS (Song, 2007). However, due to the complexity of chemical compositions of BMD and pathological processes of IS, the underlying mechanisms of BMD has still not been elucidated.

In the present study, the protective effects and related mechanisms of BMD on IS were performed *via* pharmacodynamic and metabolomic strategies, respectively. By using the middle cerebral artery occlusion–reperfusion injury (MCAO/R) rat model and UHPLC-QTOF-MS/MS technique, the neuroprotective effects of BMD on MCAO/R rats were estimated, the differential metabolites of rat’s serum and brain were employed using metabolomic analysis, and the possible mechanisms which were related to antioxidant, amino acid, and glycerophospholipid metabolisms were elucidated.

## 2 Materials and methods

### 2.1 Materials and reagents

*Eugenia caryophyllata* Thunb (EC), *Myristica fragrans* Houtt (MF), *Moschus berezovskii* Flerov (MB), and *Crocus sativus* L (CS), which were identified by Dr. Lin Dong (Department of Pharmacognosy, Ningxia Medical University), were purchased from Ming De Chinese Herbal Pieces Co., Ltd. (Ningxia, China), and the specimens (#20150701, #20150703, #20150704, and #20150705) were preserved in the Department of Pharmacy, Ningxia Medical University. Nimodipine was provided by Bayer Healthcare Co., Ltd. (Germany); nitric oxide (NO, 20181221), super oxide dismutase (SOD, 20181221), malondialdehyde (MDA, 20190308), glutathione (GSH, 20181218), and glutathiol (GSSG, 20190306) reagent kits were offered by the Institute of Nanjing Jiancheng Biological Engineering (Nanjing, China); antibodies of vascular endothelial growth factor (VEGF, ab32152), caspase-3 (ab32351), and nuclear factor-κB (NF-κB, ab32536) were obtained from Abcam Co., Ltd (Shanghai, China); acetonitrile, methanol, isopropanol, and formic acid of HPLC grade were obtained from Fisher Scientific (Nepean, Canada) and Sigma-Aldrich (St. Louis, United States); ultrapure water was obtained from Watsons Water (Guangzhou, China); and triphenyltetrazolium chloride (TTC) was obtained from Suolaibao Engineering (Beijing, China). All the other reagents were of analytical grade.

### 2.2 Preparation of BMD

BMD was prepared according to the records of the Huihui Medical Formulary and clinic. Volumes of 225 g EC and 750 g MF were powdered, mixed fully, and extracted three times by using the reflux method with 75% ethanol for 2 h each time. Then, the extractions were filtrated, merged, and ethanol was removed under reduced pressure concentrate below 60°C to obtain extracts. Finally, the extracts were fully mixed with powdered MB (7.5 g) and CS (225 g) to obtain BMD extract. For animal experiments, BMD extract was suspended in 0.5% carboxymethyl cellulose sodium (CMC-Na) aqueous solution and orally administered to rats.

### 2.3 Experimental animals and MCAO/R model establishment

All animal experiments were conducted in accordance with the laboratory animal principles and were carried out under the guidelines of the Bioethics Committee of Ningxia Medical University (license number: NXMU-2018092). A total of 90 adult male Sprague–Dawley rats (220–250 g) were provided by the Laboratory Animal Center of Ningxia Medical University (animal certification number: SCXK (Ning) 2015-0001). All animals were acclimated for 1 week in regulated polypropylene cages (three rats/cage) and standard specific pathogen-free environment (24.0 ± 0.5°C, 50%–60% humidity) with light and dark illumination cycles (12 h: 12 h). Rodent chow and water were given *ad libitum*. Then, the rats were randomly divided into six groups with 15 rats in each group: the sham operation group (SHAM, vehicle), MCAO/R model group (MCAO/R, vehicle), positive control group (NMDP, 36 mg/kg/day nimodipine), BMD low-dosage group (BMDL, 0.805 g/kg/day), BMD moderate-dosage group (BMDM, 1.61 g/kg/day), and BMD high-dosage group (BMDH, 3.22 g/kg/day). The ratio of MF:EC:CS:MB in BMD was 10:3:3:0.1, and 0.5% CMC-Na was used as a vehicle with an equal volume (100 g/ml body-weight) for each rat. All animals were administered orally once per day for 14 consecutive days.

After 14 days of prophylactic intervention, the MCAO/R-induced IS model was performed according to Nagasawa and Kogure (1989). Briefly, the rats were first anesthetized with 7% chloral hydrate (100 mg/kg, i.p.), and then the right common carotid artery (CCA), internal carotid artery (ICA), and external carotid artery (ECA) were exposed and isolated from subcutaneous muscle tissue and fascia through a neck incision (1.5–2 cm) with the CCA and ICA temporarily clipped with arterial clipping; a nylon monofilament suture (0.2 mm) with a slightly marked and rounded tip was inserted into the ECA and gently advanced from the ICA to the opening of the middle cerebral artery. The distance from the bifurcation of the ECA to the tip of the suture ranged from 18–20 mm. After 2 h of sustained MCAO, reperfusion was performed by the inserted nylon monofilament structure pulled out to restore the blood flow, and the time of reperfusion was 24 h. The SHAM operation group rats received the same surgical procedures except that the occluding monofilament was not inserted. All of the aforementioned procedures were performed in a sterile environment.

### 2.4 Neurological deficit measurements

The scores of neurological deficit of each rat was evaluated by employing Longa’s five-level neurological severity score, 24 hours after reperfusion: 0, no deficit; 1, failure to extend the right paw; 2, circling to the contralateral side uncontrollably; 3, falling to the contralateral side freely; and 4, unable to walk spontaneously or loss of consciousness.

### 2.5 Infarct volume measurement

Cerebral infarct volume was determined by using the 2,3,5-triphenyltetrazolium chloride (TTC) staining method. The rats were anesthetized, the blood samples were obtained from the femoral artery, the brain was removed immediately and cut into five coronal slices averagely, and then the slices were immersed in 2% TTC for 45 min at 37°C in the dark and turned them every 15 min. The results were analyzed by measuring the normal (red) and infarct volume ratio of the brain tissue (white). The cerebral infarction volume ratio was calculated using the following formula: the infarcted volume of the brain/the total volume of the brain × 100%.

### 2.6 Biochemical, histopathological, and immunohistochemical examinations

The levels of oxidative stress-related index including NO, SOD, MDA, GSH, and GSSG were analyzed by employing the corresponding reagent kits. For histopathological examination, the brain tissue was fixed in 4% paraformaldehyde for 12 h, and then the brain slices were dehydrated, paraffin-embedded, sectioned, and stained with hematoxylin and eosin (HE). For immunohistochemical examination, the brain slices were dehydrated, paraffin-embedded, and sectioned, and then the levels of VEGF, caspase-3, and NF-κB were determined by using a microscope. Image analysis software was used to extract details from the images.

### 2.7 Metabolomic analysis

#### 2.7.1 Sample preparation

Serum samples were obtained by centrifuging the blood samples at 4,000 rpm for 15 min, and the supernatants were collected, whereas brain tissues were placed in normal saline (NS) to remove the residual blood, and then the serum and brain samples were frozen at −80°C until analysis.

For metabolomic analysis, serum and brain samples were first re-dissolved at ambient temperature before preparation; 100 μL of each serum sample was added to 600 μL of acetonitrile, vibrated for 3 min, and centrifuged at 13,000 rpm for 10 min at 4°C, and then the supernatant was collected. The fully thawed brain was weighed precisely, and an equal weight of NS was added and grinded to obtain brain tissue homogenate. Then, 100 μL of each brain homogenate was added to 200 μL of methanol–acetonitrile solution (1:1), vibrated for 3 min, and centrifuged at 13,000 rpm for 10 min at 4°C, and the supernatant was collected. Ultimately, 1 μL aliquot of the supernatant of serum and brain samples was directly injected to UHPLC-QTOF-MS/MS for data acquisition. Meanwhile, the serum and brain quality control (QC) samples were, respectively, prepared by taking 10 μL of each sample and mixing the aliquots together, and then the QC samples were injected at regular intervals (every 10 samples) throughout the analytical run to monitor the stability from the beginning to ending of the analysis.

#### 2.7.2 UHPLC-QTOF-MS/MS conditions

UHPLC analysis was performed on an Agilent 1290 UHPLC system using a ZORBAX RRHD Eclipse Plus C_18_ column (50 mm × 2.1 mm, 1.8 μm), and the mobile phase consisted of A (0.1% formic acid in water) and B (0.1% formic acid in acetonitrile), with a gradient elution as follows: 0–1 min: 99–80% A; 1–5 min: 80–50% A; 5–10 min: 50–30% A; 10–15 min: 30–15% A; 15–16 min: 15–1% A; 16–17.5 min: 1% A; 17.5–18.5 min: 1–99% A; and 18.5–20 min: 99% A. The column temperature was maintained at 35°C, and flow rate was 0.2 ml/min. Mass spectrometry (MS) analysis was performed on an Agilent 6545 Q-TOF instrument by employing an electrospray ionization (ESI) source in both positive and negative modes. The ionization source conditions were set as follows: capillary voltage of 4.0 kV; source temperature of 120°C; desolvation temperature of 350°C; sampling cone voltage of 65 V; trap collision energy of 6.0 V; trap gas flow of 12 ml/min; atomizer pressure of 241.3 kPa; fragmentor of 130 V; and spray pressure of 40 psi; the data were collected from *m/z* 100 Da–3,000 Da.

#### 2.7.3 Data processing and pattern recognition analysis

The original datasets of all samples were first converted into *m/z*. The data were formatted using Agilent MassHunter Qualitative software and then imported to the R software package for peak detection, identification, alignment, and normalization. The retention time, peak area, and *m/z* data of each peak were collected. SIMCA (version 14.1, Umetrics AB, Umea, Sweden) was adopted for multivariate statistical analysis of the normalized data matrices: principal component analysis (PCA) and orthogonal projections to latent structure discriminant analysis (OPLS-DA). OPLS-DA modeling analysis was implemented for the first principal component, and the model quality was verified by seven-fold cross validation. Following this, the permutation test was employed to change the sequence of the classified variable Y randomly and repeatedly (200 times) to obtain different random Q^2^ values, so as to further evaluate the effectiveness of the model. The variable importance in the projection (VIP) value was obtained from the OPLS-DA model. The univariate analysis *t*-test was performed by employing SPSS (18.0). Metabolite peaks with a VIP value greater than 1 and *p*-value less than 0.05 were considered significant differential metabolites. The relevant metabolic pathways were analyzed and identified using online databases, including HMDB, KEGG, and MetaboAnalyst.

### 2.8 Statistical analysis

All of the experimental results were described as the mean ± SD and were analyzed using SPSS software (version 18.0, IBM SPSS Statistics, IBM Corp., Armonk, New York, NY, United States). Data were performed using a *t*-test and one-way analysis of variance (ANOVA). The *p*-value less than 0.05 was considered statistically significant.

## 3 Results

### 3.1 Effect of BMD on cerebral infarction

**FIGURE 1:**
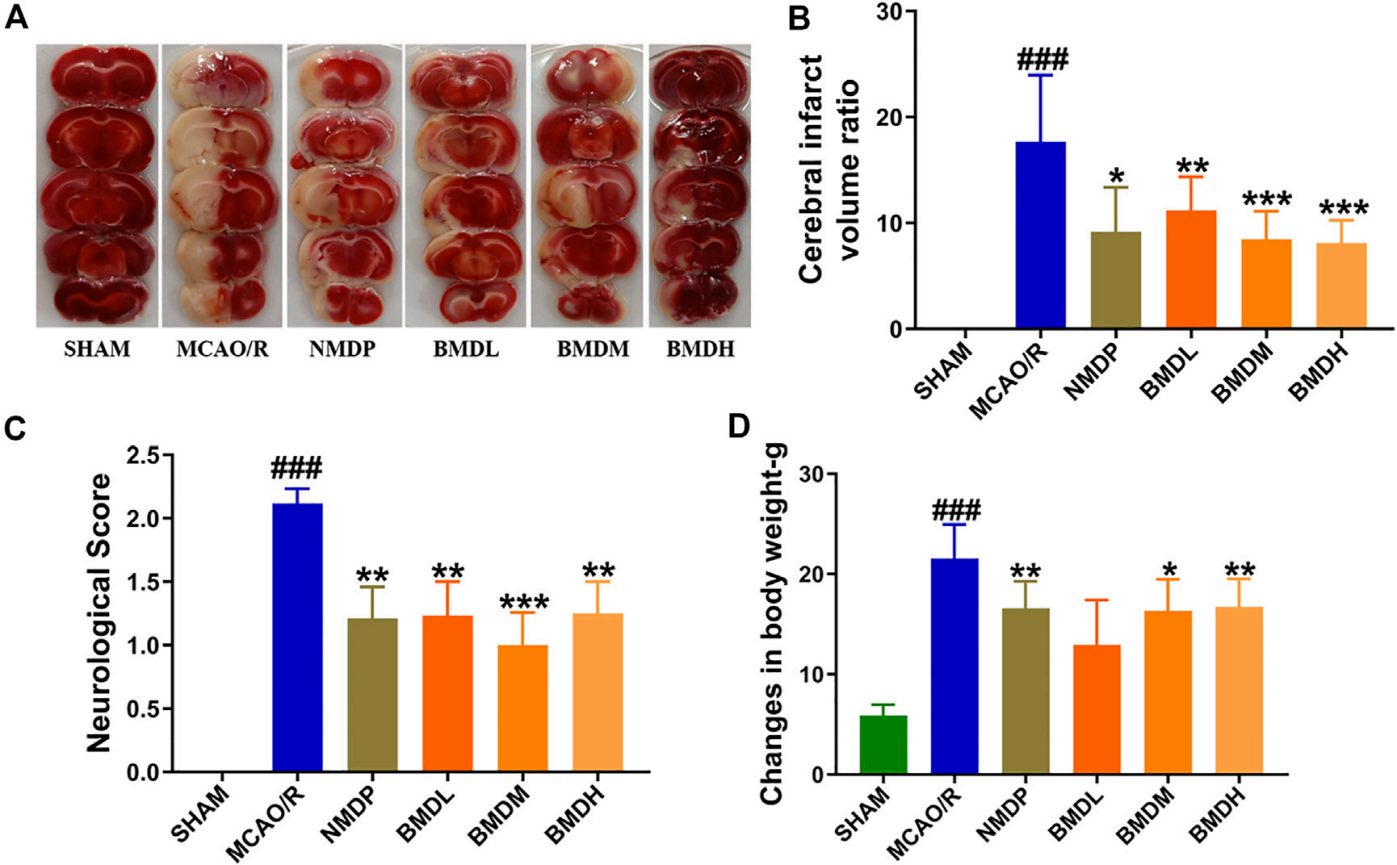
Cerebral infarction evaluation (*n* = 8). **(A)** TTC staining of brains. **(B)** Cerebral infarct volume ratio examination. **(C)** Neurological deficit score assessment. **(D)** Changes in body weight determination. Data were expressed as the mean ± SD. ^*^ *p* < 0.05, ^**^ *p* < 0.01, ^***^ *p* < 0.001 relative to the MCAO/R group; ^*###*^ *p* < 0.001 relative to the SHAM group.

Neurological deficit score and cerebral infarct volume were the two most important evaluation indexes for brain injury. As shown in Figure 1, at 24 h after reperfusion, both the neurological deficit score and cerebral infarct volume were significantly increased in the MCAO/R model group compared to the SHAM group rats (*p* < 0.001). BMD treatment markedly decreased the neurological deficit score and the cerebral infarct volume of MCAO/R rats independent of the dosage used. Moreover, to further evaluate whether BMD possessed the protective effects on MCAO/R rats, the body weight change of rats was evaluated. Despite having a similar environment after the surgery, the changes in body weight of rats in the MCAO/R group were significantly more than those in the SHAM rats (*p* < 0.001). On the contrary, these changes were all significantly reduced after BMD intervention, and the organ coefficients of the heart, liver, spleen, lung, and kidney exhibited no significant difference in all groups.

### 3.2 Effect of BMD on the markers of oxidative stress

**FIGURE 2:**
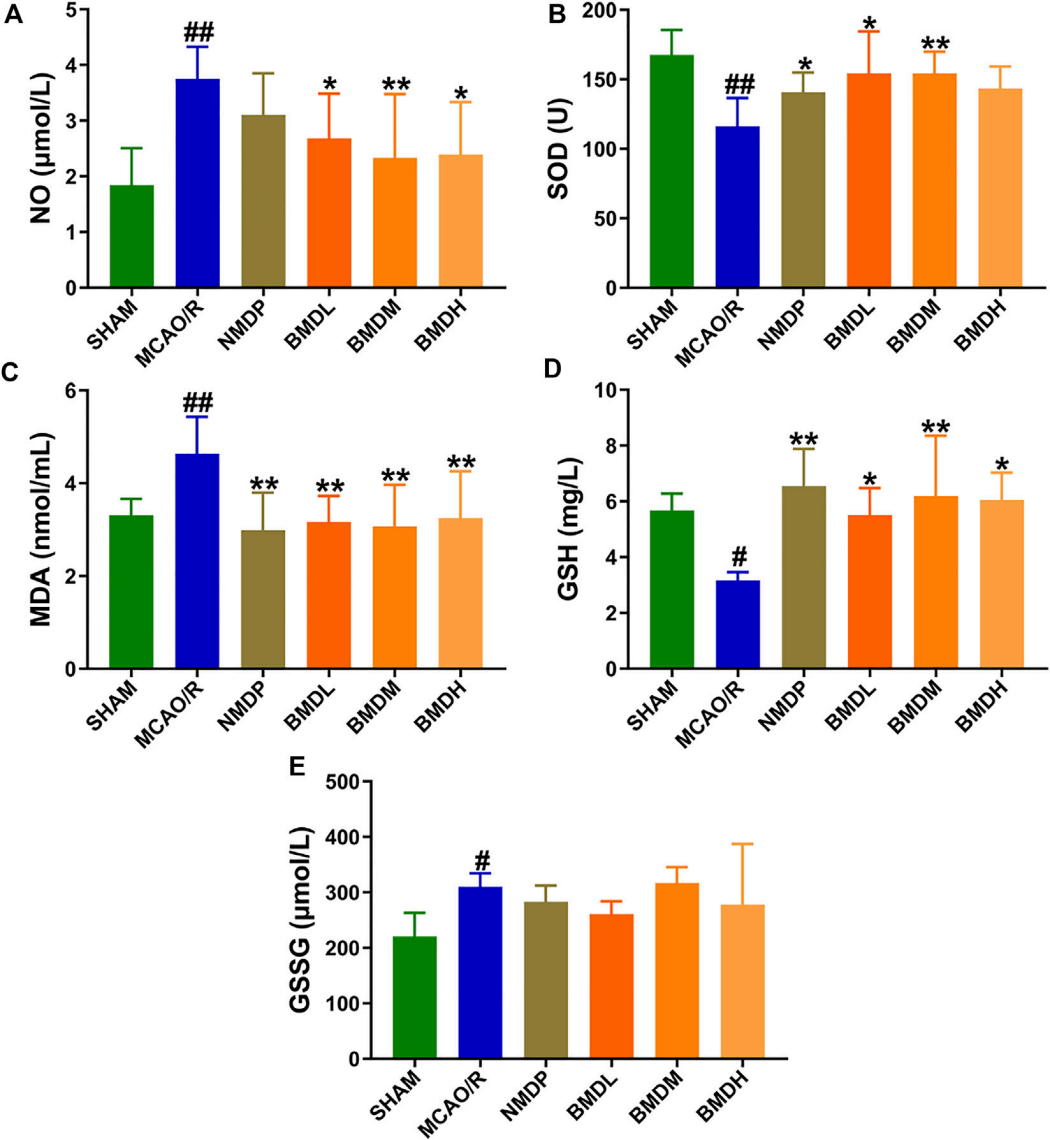
Effect of BMD on the markers of oxidative stress (*n* = 8) including NO, SOD, MDA, GSH, and GSSG. **(A)** NO. **(B)** SOD. **(C)** MDA. **(D)** GSH. **(E)** GSSG. Data were expressed as the mean ± SD. ^*^ *p* < 0.05, ^**^ *p* < 0.01 relative to the MCAO/R group; ^*#*^ *p* < 0.05, ^*##*^ *p* < 0.01 relative to the SHAM group.

The results from NO, SOD, MDA, GSH, and GSSG assessments indicated that BMD played a certain role in regulating the markers of oxidative stress. As compared with the SHAM group, the levels of serum NO, MDA, and GSSG were evidently increased, and the contents of SOD and GSH were significantly decreased (Figure 2). Concerning the NO, MDA, and GSSG levels, a declining trend (but no statistically significant difference) in the GSSG level was observed in the BMDL- and BMDH-treated groups as compared to the MCAO/R group, whereas BMD at all three dosages evidently declined the NO and MDA levels in the MCAO/R rats (*p* < 0.05). For SOD and GSH, as shown in Figure 2, significantly increased trends were obtained in the BMD intervention groups as compared to the MCAO/R model group except the high dosage of BMD which showed no significant change in the SOD level.

### 3.3 Effect of BMD on histopathological and immunohistochemical abnormality of brain tissue

**FIGURE 3:**
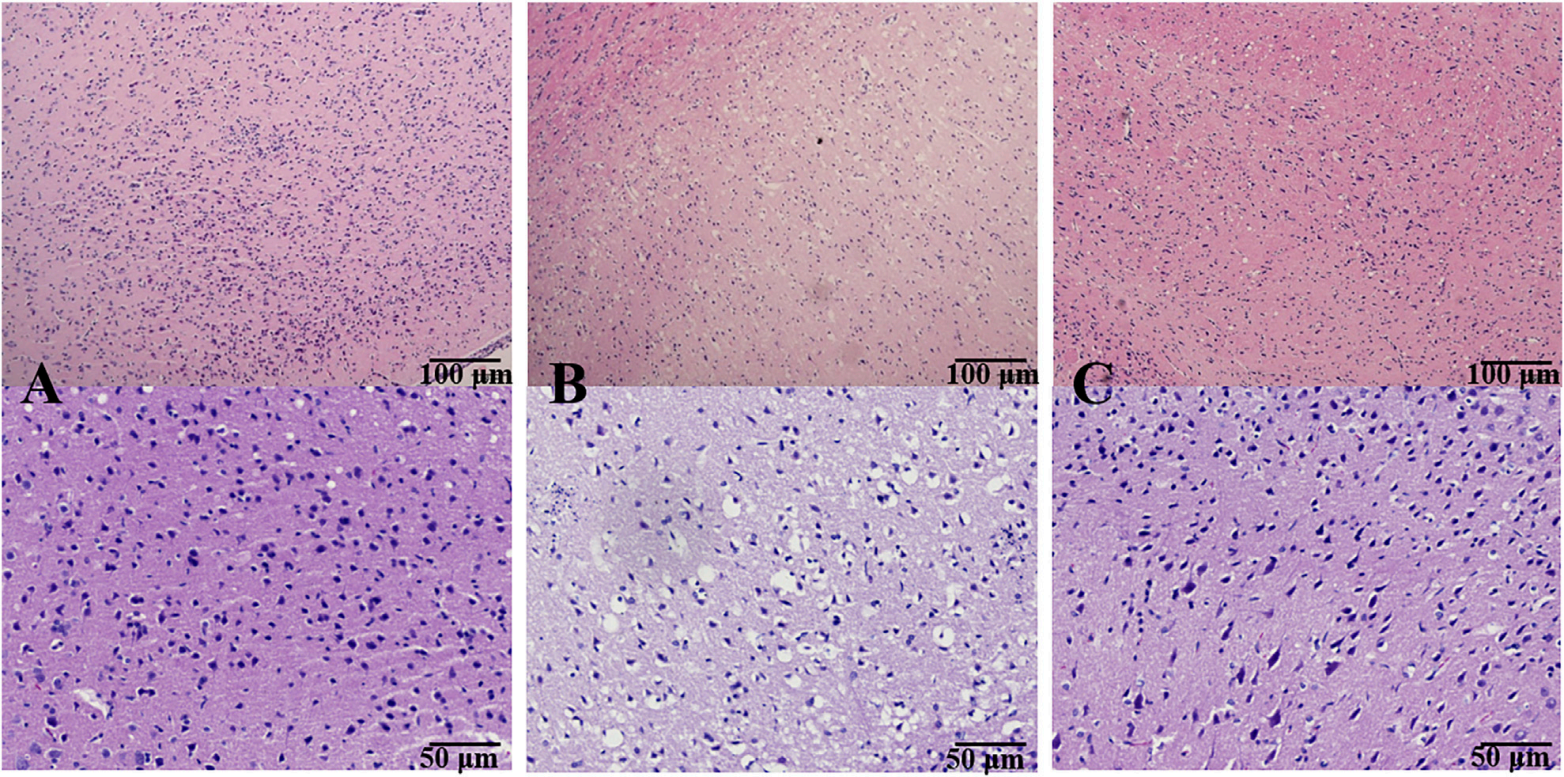
Effect of BMD on histopathological abnormality of brain tissue. Original magnification: ×4 and ×10. **(A)** SHAM group; **(B)** MCAO/R group; and **(C)** BMDM group.

Figure 3 shows the morphological features of neurons by H&E staining. In the SHAM group, the frame of the brain tissue was clear with no pathological abnormalities in the cerebral cortex. On the contrary, an obvious change was found in the MCAO/R model group, evidenced by the signs of neuronal loss, vacuolated spaces, and shrunken and disordered neurons. After the prophylactic administration, BMD remarkably ameliorated the aforementioned pathological abnormalities in the brain tissue of MCAO/R rats.

**FIGURE 4:**
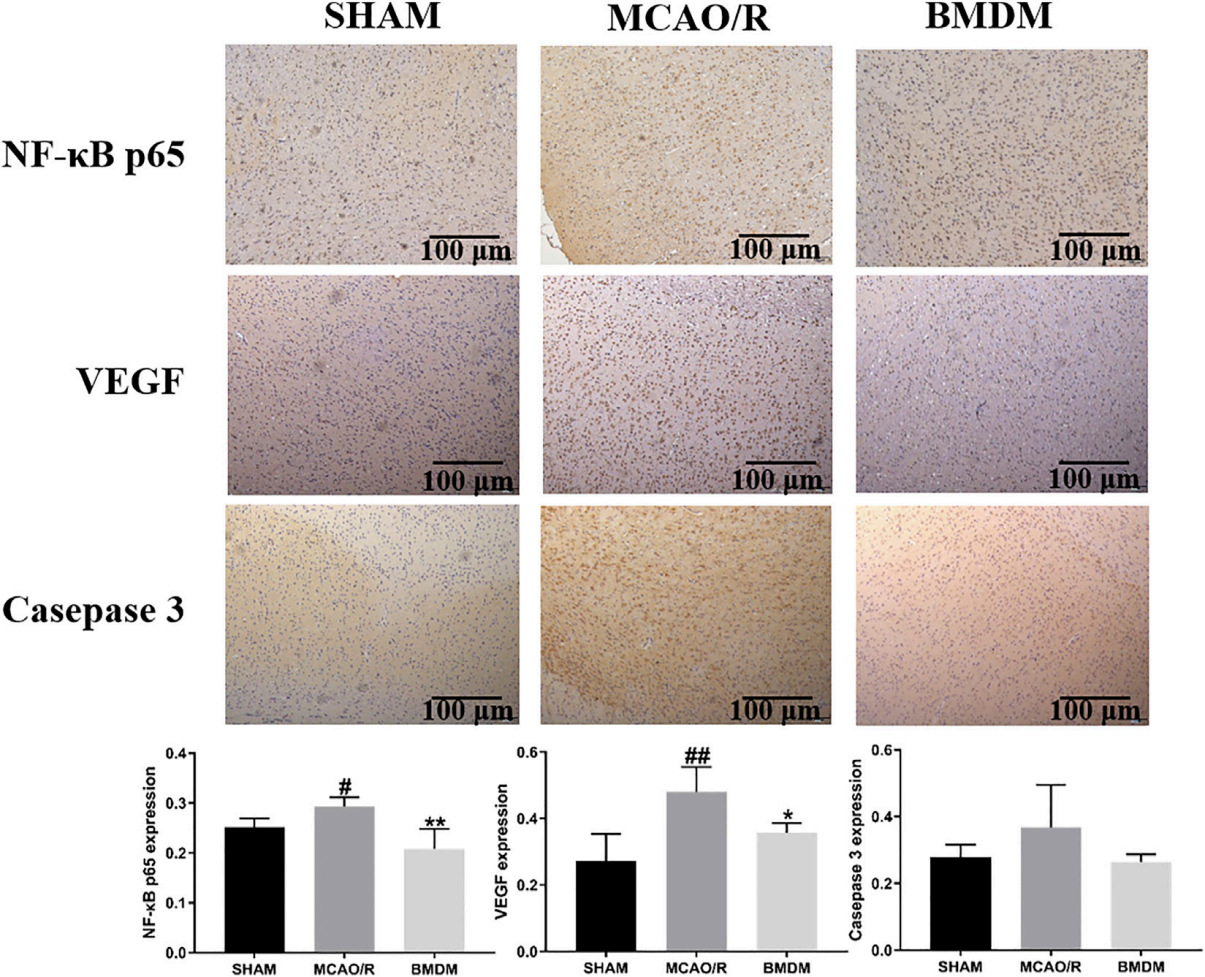
Effect of BMD on immunohistochemical abnormality of brain tissue. Original magnification: ×4. Data were expressed as the mean ± SD. ^*^ *p* < 0.05, ^**^ *p* < 0.01 relative to the MCAO/R group; ^*#*^ *p* < 0.05, ^*##*^ *p* < 0.01 relative to the SHAM group.

To further explain the neuroprotective effects of BMD on the abnormality of brain tissue, the levels of NF-κB, VEGF, and caspase-3 were detected using immunohistochemical examination. As shown in Figure 4, the expression of NF-ĸB and VEGF in the MCAO/R model group was obviously increased, and an increasing trend but no statistical significance of the caspase-3 level was observed in MCAO/R model rats as compared with SHAM rats. All BMD-treated rats showed significantly decreased NF-κB p65, VEGF, and caspase-3 levels as compared to MCAO/R model rats, indicating that less brain injury was obtained in MCAO/R rats upon BMD prophylactic administration.

### 3.4 Effect of BMD on differential metabolites

#### 3.4.1 Validation of the UHPLC-QTOF-MS/MS analysis method

To verify the repeatability and stability of the UHPLC-QTOF-MS/MS metabolomic method, the representative TICs of the serum sample in positive and negative ion modes are shown in Supplementary Figures S1, S2, respectively. The relative standard deviations (*RSD*s) of the typical peak areas and retention times were all less than 15% in positive and negative modes (Supplementary Table S1), which indicated the suitability and reliability of our metabolomic method.

#### 3.4.2 Multivariate data analysis for serum and brain samples

**FIGURE 5:**
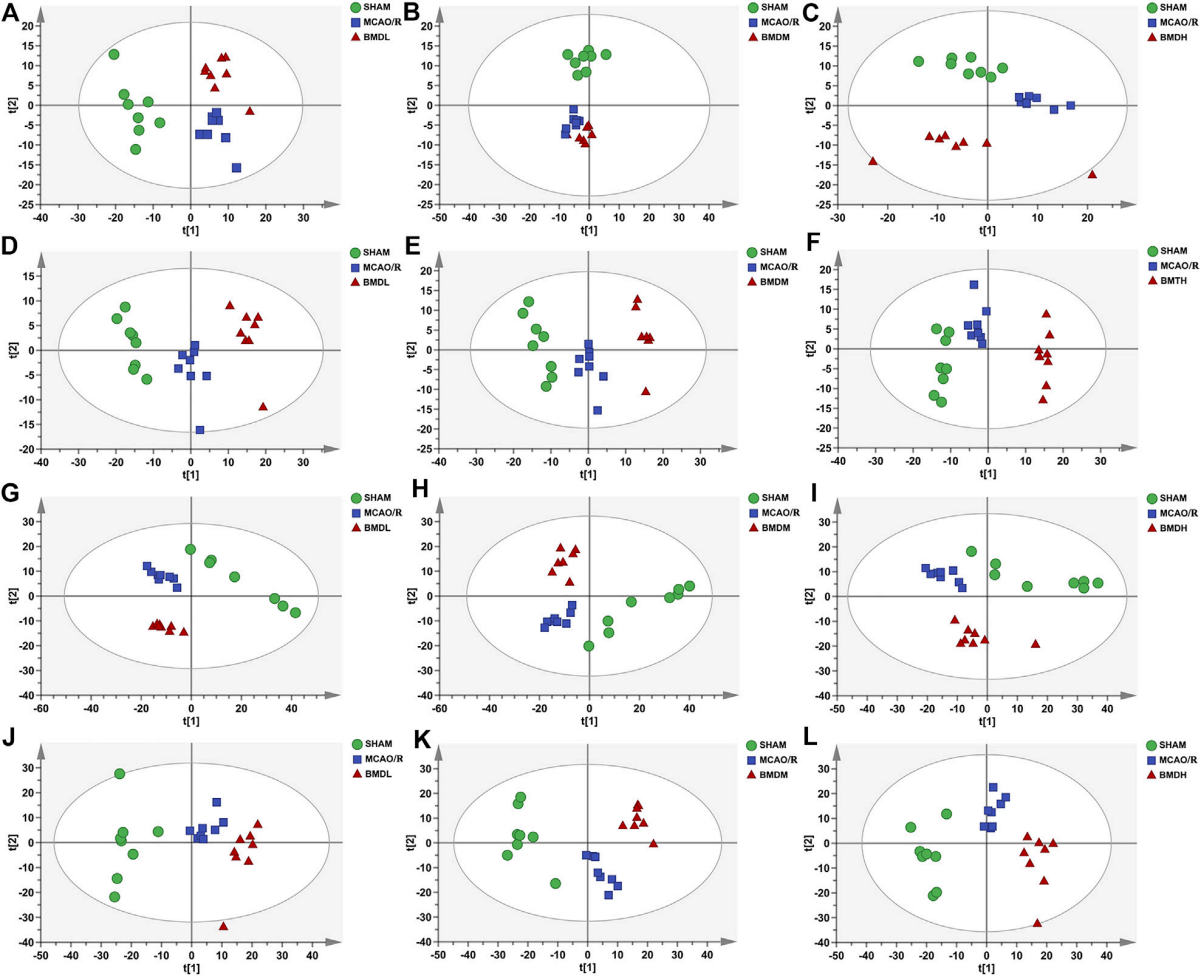
PCA score plots of serum samples from SHAM, MCAO/R, BMDL, BMDM, and BMDH in positive **(A–C)** and negative **(D–F)** ESI modes (*n* = 8); PCA score plots of brain samples from SHAM, MCAO/R, BMDL, BMDM, and BMDH in positive **(G–I)** and negative **(J–L)** ESI modes (*n* = 8).

**FIGURE 6:**
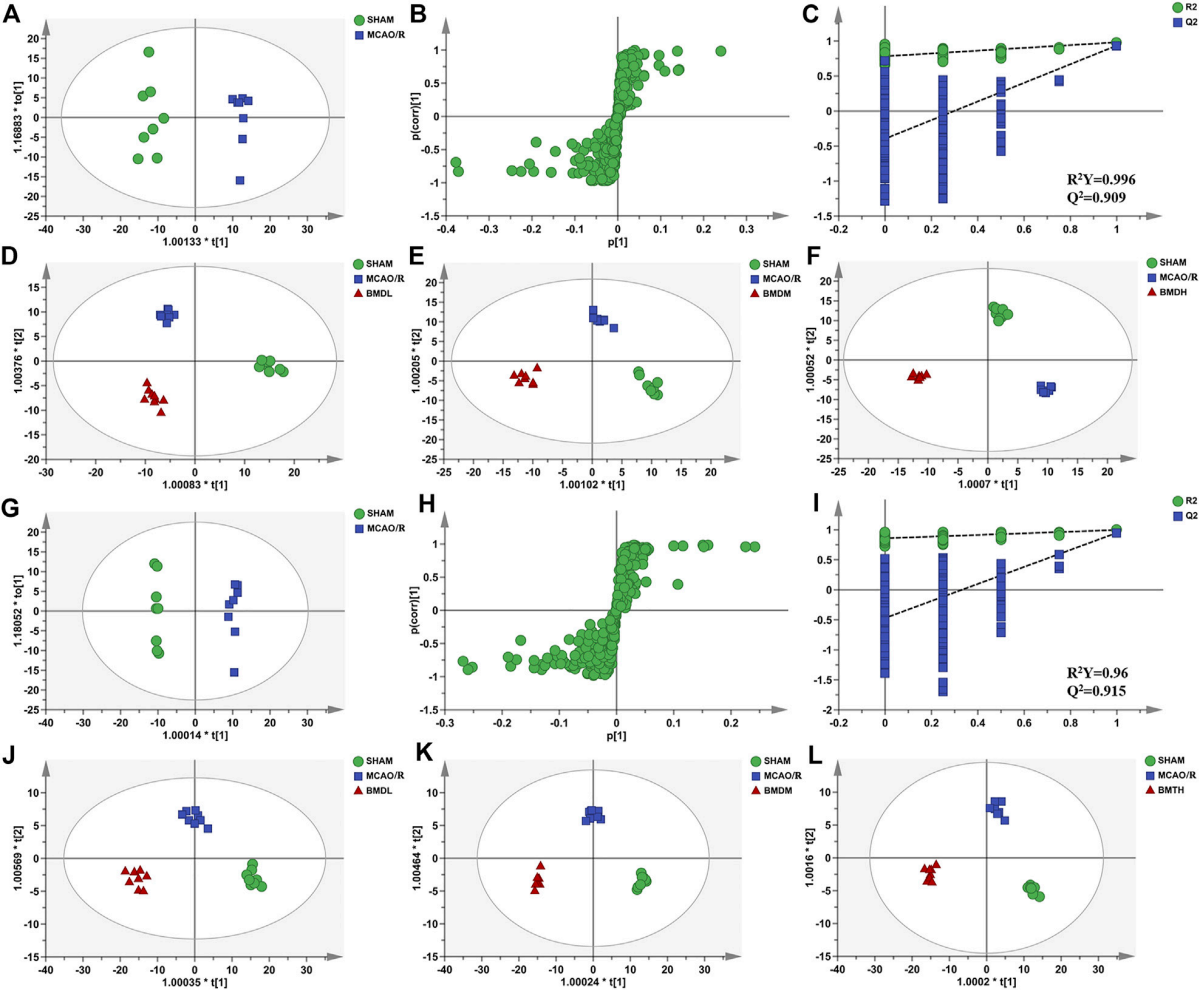
OPLS-DA score plots, S-plots, and permutation validation of serum samples. **(D–F)** OPLS-DA score plots of SHAM, MCAO/R, BMDL, BMDM, and BMDH in the positive mode; **(J–L)** OPLS-DA score plots of SHAM, MCAO/R, BMDL, BMDM, and BMDH in the negative mode; **(A,G)** OPLS-DA score plots of SHAM and MCAO/R in positive and negative modes, respectively; **(B,H)** S-plots of SHAM and MCAO/R in positive and negative modes, respectively; **(C, I)** permutation validation of SHAM and MCAO/R in positive and negative modes, respectively (*n* = 8).

**FIGURE 7:**
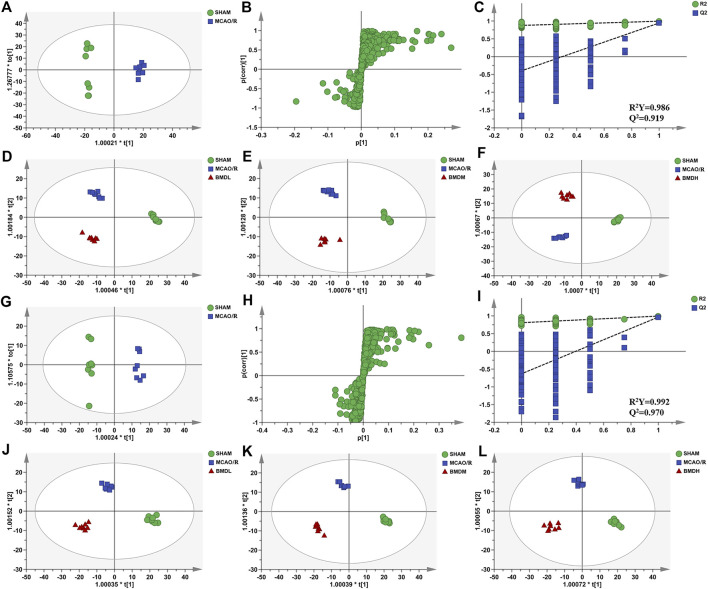
OPLS-DA score plots, S-plots, and permutation validation of brain samples. **(D–F)** OPLS-DA score plots of SHAM, MCAO/R, BMDL, BMDM, and BMDH in the positive mode; **(J–L)** OPLS-DA score plots of SHAM, MCAO/R, BMDL, BMDM, and BMDH in the negative mode; **(A,G)** OPLS-DA score plots of SHAM and MCAO/R in positive and negative modes, respectively; **(B,H)** S-plots of SHAM and MCAO/R in positive and negative modes, respectively; **(C,I)** permutation validation of SHAM and MCAO/R in positive and negative modes, respectively (*n* = 8).

In order to explore the systemic changes in the metabolome of MCAO/R rats intervened with or without BMD, both serum and brain samples were employed for UHPLC-QTOF-MS/MS metabolomic analysis, by using positive and negative ion modes. As shown in Figure 5, SHAM, MCAO/R, and BMD groups were separated from each other in both positive and negative ion modes, suggesting that the endogenous metabolites among them were different. To discriminate the potential endogenous metabolites, the OPLS-DA model was constructed to investigate the pathogenesis of IS induced by MCAO/R and the neuroprotective mechanism of BMD. The OPLS-DA results of serum and brain metabolomic profiling are shown in Figures 6, 7, respectively. Moreover, the model quality was estimated by employing R^2^Y and Q^2^ values. Correspondingly, R^2^Y and Q^2^ were all bigger than 0.9, indicating good fitness and predictability of this model.

#### 3.4.3 Potential endogenous metabolite identification and metabolic pathway analysis

**FIGURE 8:**
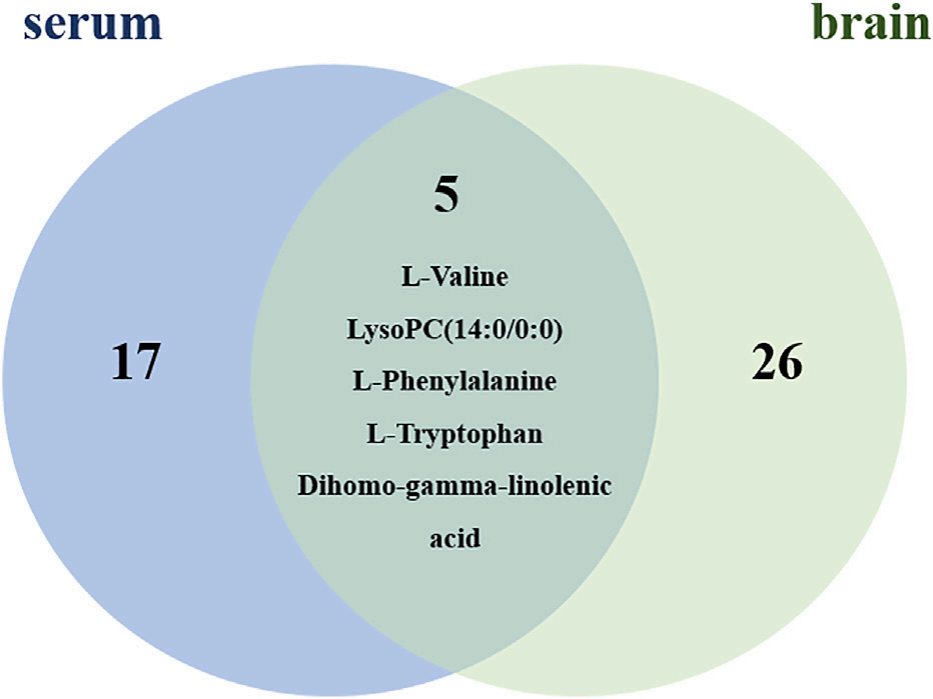
Metabolite differences between serum and brain samples.

**FIGURE 9:**
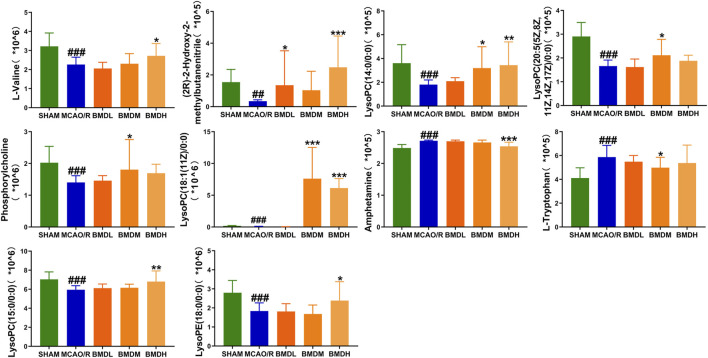
Differential metabolite changes in MCAO/R with BMD intervention in serum. Data were expressed as the mean ± SD. ^*^ *p* < 0.05, ^**^ *p* < 0.01, ^***^ *p* < 0.001 relative to the MCAO/R group; ^*##*^ *p* < 0.01, ^*###*^ *p* < 0.001 relative to the SHAM group.

**FIGURE 10:**
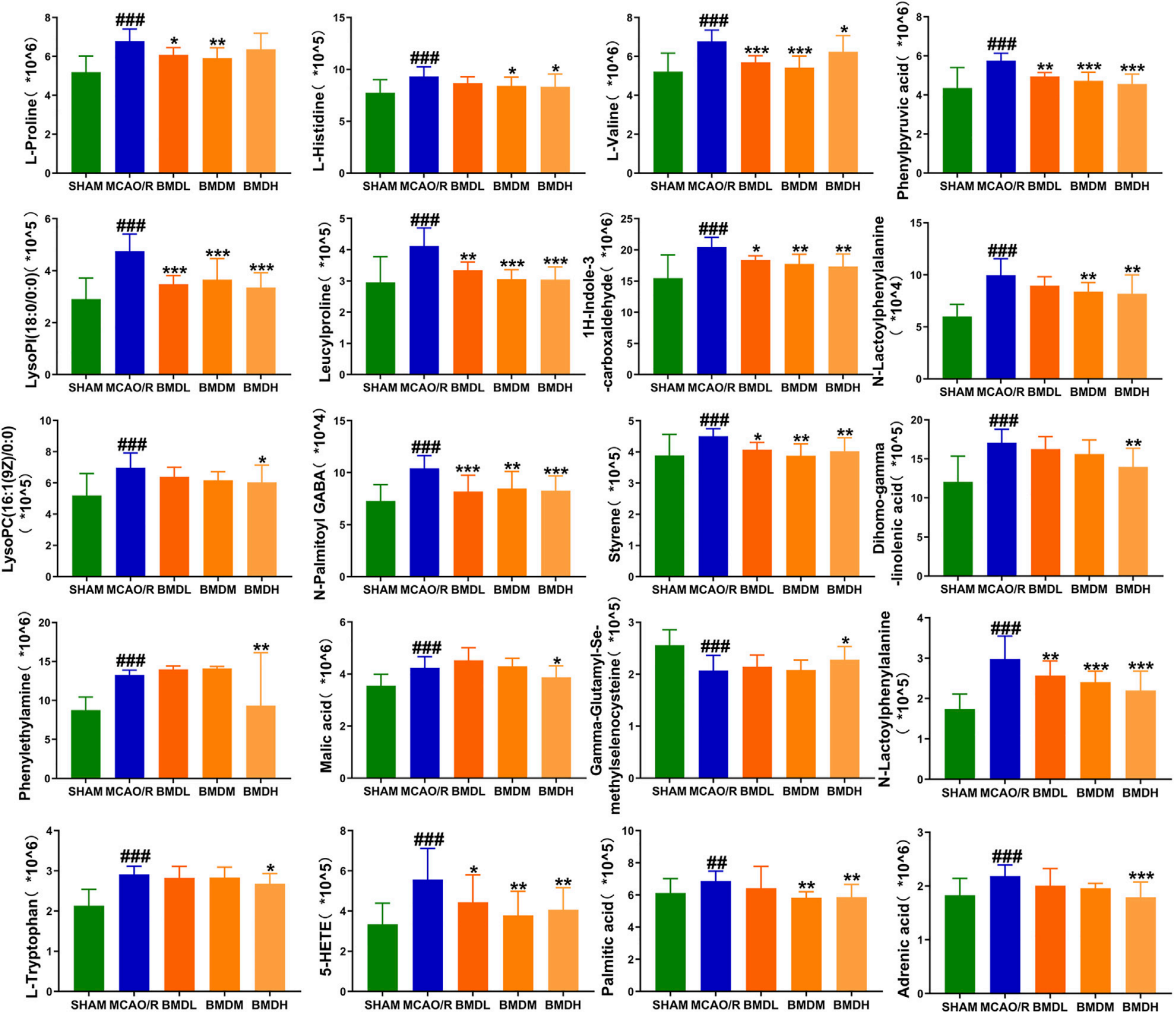
Differential metabolite changes in MCAO/R with BMD intervention in the brain. All values were presented as the mean ± SD. ^*^ *p* < 0.05, ^**^ *p* < 0.01, ^***^ *p* < 0.001 relative to the MCAO/R group; ^*##*^ *p* < 0.01, ###*p* < 0.001 relative to the SHAM group.

**FIGURE 11:**
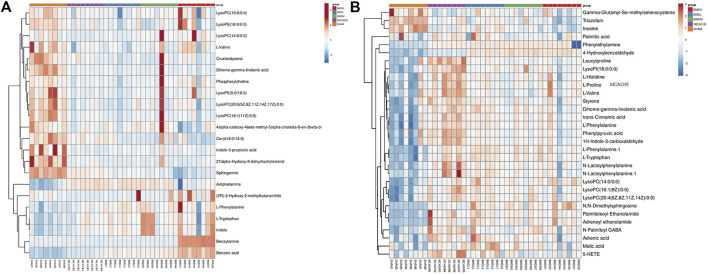
Analysis of differential metabolites in MCAO/R rats related to the curative effect of BMD. Heatmap of spearman correlation analysis between metabolites in serum **(A)** and brain **(B)** samples of all groups.

**FIGURE 12:**
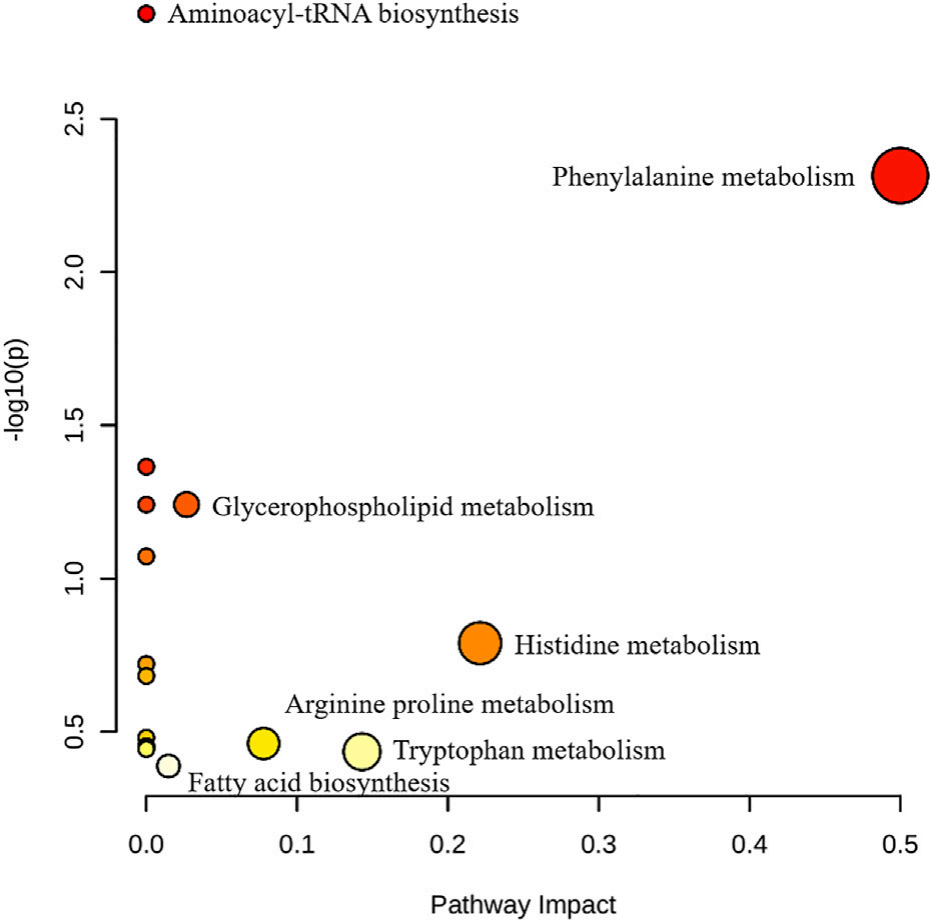
Pathway analysis of BMD intervention. The metabolic pathways involved in the protection effects of BMD on MCAO/R rats.

**FIGURE 13:**
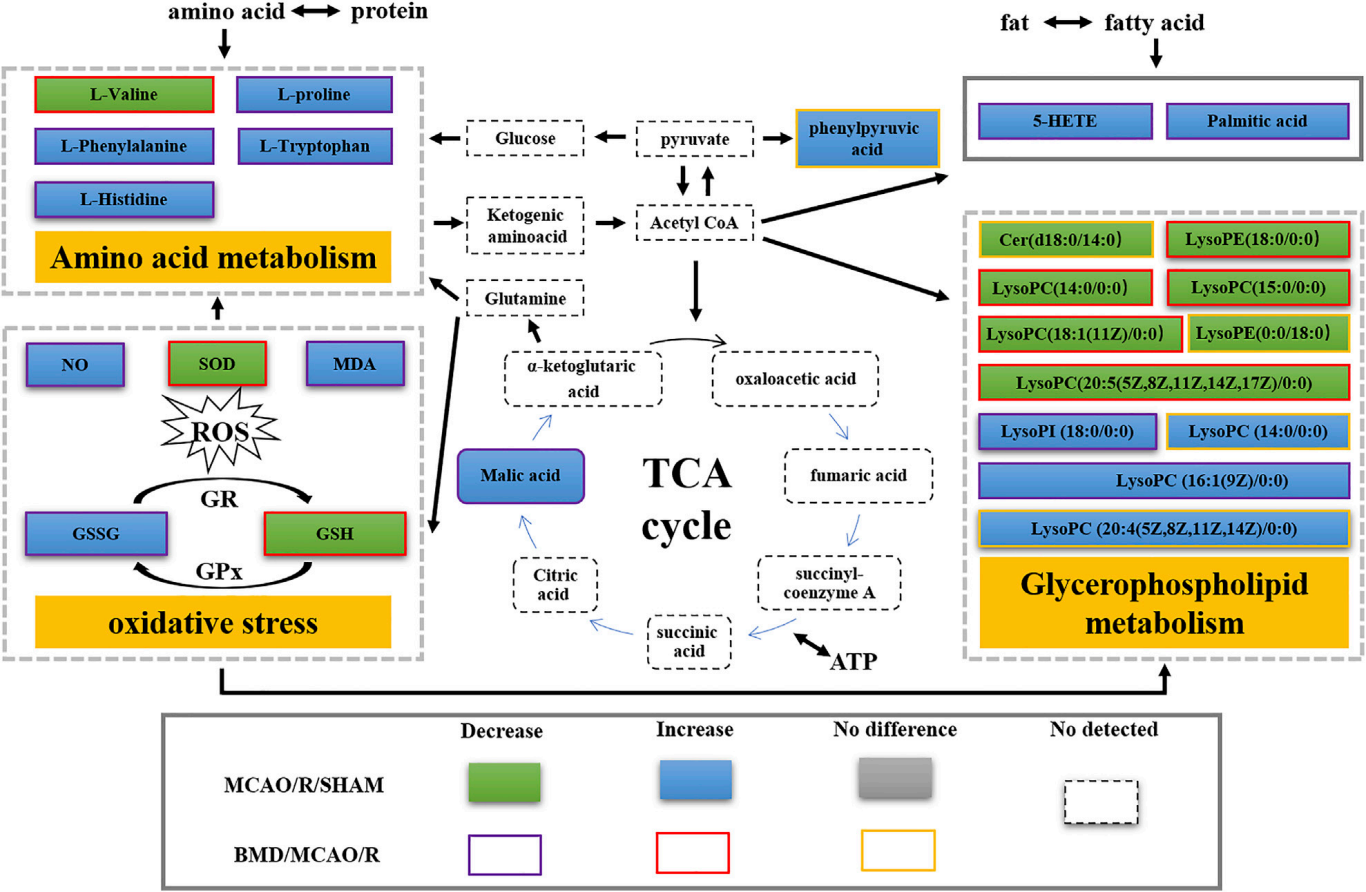
Metabolic disorder map of the MCAO/R model rats.

By using the OPLS-DA model, the differential metabolites in serum and brain samples between SHAM and MCAO/R groups associated with IS were screened. Setting the significance level at *p* < 0.05 and VIP>1, 22 differential endogenous metabolites in serum samples (Table 1) and 31 endogenous metabolites in brain samples (Table 2) were identified. Subsequently, a Venn diagram was adopted to analyze overlapping endogenous metabolites in the serum and brain samples (Figure 8), and the results showed that L-valine, lysoPC (14:0/0:0), L-phenylalanine, L-tryptophan, and dihomo-gamma-linolenic acid could be detected in both serum and brain samples. In addition, among these 53 differential endogenous metabolites, 30 metabolites in the MCAO/R rats were restored after administration of BMD, specifically 10 of them were found in serum and 20 were found in the brain (Figures 9, 10). The cluster analysis of metabolites is shown as a heatmap in Figure 11. To explore the underlying relevance between the identified metabolites involved in IS and BMD intervention, the main biological metabolic pathway analysis was performed using the MetaboAnalyst 5.0 online database based on the identified differential metabolites in serum and brain samples. The results are shown in Figure 12, where 30 metabolites restored by BMD were associated with seven main metabolisms, and they were phenylalanine metabolism, histidine metabolism, tryptophan metabolism, arginine and proline metabolism, glycerophospholipid metabolism, fatty acid biosynthesis, and aminoacyl-tRNA biosynthesis. The mechanism analysis of the differential metabolites with the corresponding pathways is shown in Figure 13.

**TABLE 1:** Statistical analysis results of identified metabolite changes in serum.

No.	Adduct	Name	Formula	M/Z	RT/min	HMDB	Fold	*p*-value	VIP	MCAO/R vs. SHAM	Pathway
1	M + H	L-Valine	C_5_H_11_NO_2_	118.0861	0.735	HMDB0000883	1.3890	6.91E-05	1.3136	Down	Amino acid metabolism
2	M + H	(2R)-2-Hydroxy-2-methylbutanenitrile	C_5_H_9_NO	100.0759	1.897	HMDB0060309	4.0741	6.12E-05	1.3693	Down	Cyanoamino acid metabolism
3	M + H	Indole-3-propionic acid	C_11_H_11_NO_2_	190.0868	4.484	HMDB0002302	4.9225	3.79E-05	1.4864	Down	Amino acid metabolism
4	M + H	LysoPC (14:0/0:0)	C_22_H_46_NO_7_P	468.3086	7.313	HMDB0010379	2.0701	3.88E-04	1.2378	Down	Glycerophospholipid metabolism
5	M + H	LysoPC (20:5 (5Z,8Z,11Z,14Z,17Z)/0:0)	C_28_H_48_NO_7_P	542.3218	7.654	HMDB0010397	1.7305	5.61E-07	1.5602	Down	Glycerophospholipid metabolism
6	M + H	LysoPE (0:0/18:0)	C_23_H_48_NO_7_P	482.3246	8.071	HMDB0011129	2.4997	9.88E-06	1.4567	Down	Glycerophospholipid metabolism
7	M + Na	Crustecdysone	C_27_H_44_O_7_	503.2980	8.303	HMDB0030180	2.1569	4.33E-06	1.4408	Down	Fatty acids and their derivatives
8	M + H	Phosphorylcholine	C_5_H_15_NO_4_P	184.0736	8.624	HMDB0001565	1.4665	3.97E-04	1.2127	Down	Glycerophospholipid metabolism
9	M + H	LysoPC (18:1 (11Z)/0:0)	C_26_H_52_NO_7_P	522.3563	9.423	HMDB0010385	2.2128	7.52E-06	1.4701	Down	Glycerophospholipid metabolism
10	M + H	Dihomo-gamma-linolenic acid	C20H34O2	307.2635	14.700	HMDB0002925	2.4043	1.15E-07	1.6767	Down	Glycerophospholipid metabolism
11	M + H	Cer (d18:0/14:0)	C_32_H_65_NO_3_	512.5030	16.348	HMDB0011759	1.2193	1.65E-03	1.1095	Down	Fatty acid metabolism
12	M + H	27α-Hydroxy-8-dehydrocholesterol	C_27_H_44_O_2_	401.3417	17.518	HMDB0060132	3.7049	7.17E-07	1.5677	Down	Steroid biosynthesis
13	M + H	4α-Carboxy-4β-methyl-5α-cholesta-8-en-3β-ol	C_29_H_48_O_3_	445.3677	17.567	HMDB0062384	1.5022	2.16E-04	1.2293	Down	Cholesterol metabolism
14	M + H	Amphetamine	C_9_H_13_N	136.1123	18.792	HMDB0014328	1.0853	1.63E-07	1.6397	Up	Pyrimidine metabolism
15	M + H	Sphinganine	C_18_H_39_NO_2_	302.3058	18.858	HMDB0000269	1.2181	6.09E-07	1.5410	Down	Glycerophospholipid metabolism
16	M + H	Benzylamine	C_7_H_9_N	108.0809	18.877	HMDB0033871	1.1455	2.61E-11	1.7798	Up	Amino acid metabolism
17	M-H	L-Phenylalanine	C_9_H_11_NO_2_	164.0714	1.843	HMDB0000159	1.3774	8.37E-06	1.1886	Up	Amino acid metabolism
18	M-H	L-Tryptophan	C_11_H_12_N_2_O_2_	203.0820	2.186	HMDB0000929	1.3389	3.70E-04	1.0230	Up	Amino acid metabolism
19	M-H	Indole	C_8_H_7_N	116.0509	2.190	HMDB0000738	1.3857	4.71E-05	1.1046	Up	Amino acid metabolism
20	M-H	Benzoic acid	C_7_H_6_O_2_	121.0291	2.551	HMDB0001870	1.1426	3.73E-08	1.4073	Up	Amino acid metabolism
21	M-H	LysoPC (15:0/0:0)	C_23_H_48_NO_7_P	480.3093	8.907	HMDB0010381	1.1617	1.47E-04	1.0301	Down	Glycerophospholipid metabolism
22	M-H	LysoPE (18:0/0:0)	C_23_H_48_NO_7_P	480.3090	10.735	HMDB0011130	1.5225	4.62E-06	1.1642	Down	Glycerophospholipid metabolism

**TABLE 2:** Statistical analysis results of identified metabolite changes in the brain.

No.	Adduct	Name	Formula	M/Z	RT/min	HMDB	Fold	*p*-value	VIP	MCAO/R vs. SHAM	Pathway
1	M + H	L-Proline	C_5_H_9_NO_2_	116.0709	0.756	HMDB0000162	1.3049	0.0000	1.2571	Up	Amino acid metabolism
2	M + H	L-Histidine	C_6_H_9_N_3_O_2_	156.0767	0.772	HMDB0000177	1.2038	0.0002	1.0146	Up	Amino acid metabolism
3	M + H	L-Valine	C_5_H_11_NO_2_	118.0867	0.858	HMDB0000883	1.2979	0.0000	1.2011	Up	Amino acid metabolism
4	M + H	Phenylpyruvic acid	C_9_H_8_O_3_	165.0549	1.466	HMDB0000205	1.3202	0.0000	1.1671	Up	Phenylpyruvic acid metabolism
5	M + H	trans-Cinnamic acid	C_9_H_8_O_2_	149.0595	1.955	HMDB0000930	1.2411	0.0000	1.1885	Up	Amino acid metabolism
6	M + H	L-Phenylalanine	C_9_H_11_NO_2_	166.0864	1.955	HMDB0000159	1.2343	0.0001	1.1657	Up	Amino acid metabolism
7	M + H	LysoPI (18:0/0:0)	C_27_H_53_O_12_P	601.3344	2.096	HMDB0240261	1.6325	0.0000	1.3573	Up	Glycerophospholipid metabolism
8	M + H	Leucylproline	C_11_H_20_N_2_O_3_	229.1549	2.146	HMDB0011175	1.3933	0.0000	1.1037	Up	Amino acid metabolism
9	M + H	1H-Indole-3-carboxaldehyde	C_9_H_7_NO	146.0602	2.228	HMDB0029737	1.3225	0.0000	1.1782	Up	Amino acid metabolism
10	M + H	N-Lactoylphenylalanine	C_12_H_15_NO_4_	238.1077	3.276	HMDB0062175	1.6622	0.0000	1.3904	Up	Amino acid metabolism
11	M + H	LysoPC (14:0/0:0)	C_22_H_46_NO_7_P	468.3088	7.314	HMDB0010379	1.4068	0.0000	1.1772	Up	Glycerophospholipid metabolism
12	M + H	LysoPC (16:1 (9Z)/0:0)	C_24_H_48_NO_7_P	494.3243	7.813	HMDB0010383	1.3400	0.0002	1.1085	Up	Glycerophospholipid metabolism
13	M + H	LysoPC (20:4 (5Z,8Z,11Z,14Z)/0:0)	C_28_H_50_NO_7_P	544.3404	8.384	HMDB0010395	1.3553	0.0003	1.0765	Up	Glycerophospholipid metabolism
14	M + H	Palmitoleoyl ethanolamide	C_18_H_35_NO_2_	298.2744	10.735	HMDB0013648	3.2995	0.0000	1.2799	Up	Fatty acid metabolism
15	M + H	N-Palmitoyl GABA	C_20_H_39_NO_3_	342.3006	12.848	HMDB0062338	1.4290	0.0000	1.2852	Up	Phospholipid metabolism
16	M + H	Adrenoyl ethanolamide	C_24_H_41_NO_2_	376.3216	13.470	HMDB0013626	6.8239	0.0000	1.2598	Up	Phospholipid metabolism
17	M + H	Styrene	C_8_H_8_	105.0699	14.651	HMDB0034240	1.1586	0.0021	1.0463	Up	Ethylbenzene degradation
18	M + H	N, N-Dimethylsphingosine	C_20_H_41_NO_2_	328.3216	15.218	HMDB0013645	11.7197	0.0000	1.4420	Up	Phospholipid metabolism
19	M + H	Dihomo-gamma-linolenic acid	C_20_H_34_O_2_	307.2637	15.717	HMDB0002925	1.4151	0.0000	1.2222	Up	Linoleic acid metabolism
20	M + H	Phenylethylamine	C_8_H_11_N	122.0967	18.716	HMDB0012275	1.5167	0.0000	1.4788	Up	Amino acid metabolism
21	M-H	Malic acid	C_4_H_6_O_5_	133.0148	0.821	HMDB0000744	1.1945	2.31E-05	1.1197	Up	Pyruvate metabolism
22	M-H	Gamma-glutamyl-Se-methylselenocysteine	C_9_H_16_N_2_O_5_Se	311.0157	0.839	HMDB0010716	1.2357	1.47E-05	1.2170	Down	Selenocompound metabolism
23	M-H	Triazolam	C_17_H_12_Cl_2_N_4_	341.0363	1.476	HMDB0015034	1.2225	6.92E-06	1.2027	Down	Neuroactive ligand–receptor interaction
24	M-H	Inosine	C_10_H_12_N_4_O_5_	267.0734	1.718	HMDB0000195	1.2029	1.12E-05	1.1971	Down	Amino acid metabolism
25	M-H	L-Phenylalanine	C_9_H_11_NO_2_	164.0716	1.942	HMDB0000159	1.4046	3.00E-09	1.4346	Up	Amino acid metabolism
26	M-H	N-Lactoylphenylalanine	C_12_H_15_NO_4_	236.0926	2.009	HMDB0062175	1.7126	1.13E-08	1.4373	Up	Amino acid metabolism
27	M-H	L-Tryptophan	C_11_H_12_N_2_O_2_	203.0825	2.202	HMDB0000929	1.3637	5.81E-08	1.3820	Up	Amino acid metabolism
28	M-H	4-Hydroxybenzaldehyde	C_7_H_6_O_2_	121.0294	2.552	HMDB0011718	1.1015	3.97E-06	1.2443	Up	Hydroxybenzaldehyde
29	M-H	5-HETE	C_20_H_32_O_3_	319.2274	10.186	HMDB0011134	1.6689	1.77E-05	1.2122	Up	Arachidonic acid
30	M-H	Palmitic acid	C_16_H_32_O_2_	255.2325	16.203	HMDB0000220	1.1220	5.70E-03	1.0388	Up	Fatty acid metabolism
31	M-H	Adrenic acid	C_22_H_36_O_2_	331.2640	16.484	HMDB0002226	1.1945	2.57E-04	1.0614	Up	Biosynthesis of unsaturated fatty acid

## 4 Discussion

Stroke is a serious medical emergency worldwide, ranking second only to cancer as a cause of death (Ouk et al., 2019). IS, transient IS, and hemorrhagic stroke are the three types of stroke, and among them, IS was thought to be generally caused by vascular obstruction, which brings out severe nerve injury (Chen et al., 2021). Owing to its high mortality and disability, an early diagnosis and intervention are of great importance to decline the damage of brain tissue (Yang et al., 2021).

BMD is a classic TCM formula, which was traditionally used to relieve physical weakness and improve facial paralysis of IS patients. To confirm the protective effects of BMD, the main pharmacodynamic features such as neurological deficit score, cerebral infarct volume, and body weight change of MCAO/R rats were analyzed, and all of the results proved the protective efficacy of BMD on IS. Biochemical index examination revealed that BMD could regulate the levels of NO, SOD, MDA, GSH, and GSSG, consequently alleviating the oxidative stress injury. IS was defined as a pathological condition which related to a redox imbalance, the presence of oxidative stress was a great contributor to the pathophysiology of ischemia/reperfusion injuries (Chehaibi et al., 2016; Jurcau and Ardelean, 2021), and the oxidative stress was considered one of the earliest signs of brain injury (Schaller and Graf, 2004). The present study revealed that after MCAO/R surgery and during the process of ischemia, NO (the precursor of peroxynitrite) and MDA (the product of lipid peroxidation) were significantly increased, which indicated a severe oxidative damage resulted from free radical and reactive oxygen species (ROS). Meanwhile, because of the imbalance of antioxidant systems, SOD, GSH, GSH-Px, and other antioxidant enzymes could be affected by the condition of cerebral ischemia reperfusion. Similar to the published data, the levels of SOD and GSH in rats of the MCAO/R group were significantly decreased (Chehaibi et al., 2016), whereas the activity of GSSG was obviously increased as compared with the SHAM rats (Kozer et al., 2003; Pradeep et al., 2012). Furthermore, histopathological results revealed that BMD had protective effects on brain injury by reducing the occurrence of neuronal loss, vacuolated spaces, and shrunken and disordered neurons. Meanwhile, there were no obvious side effects observed on each organ or mortality, suggesting the safety of BMD.

In order to obtain a better understanding of the effects of oxidative stress on IS, immunohistochemical examination was performed in the present study, which might provide a new visual angle for the prevention of IS. NF-ĸB was proved to regulate the transcription of genes related to stress, inflammatory, and growth responses. Meanwhile, NF-ĸB could promote ROS production and inflammatory responses after ischemic reperfusion (Sivandzade et al., 2019; Li et al., 2021). VEGF, a mediator of vascular permeability, was found to play an important role in the chronic neuro-inflammation. The key antioxidant genes such as SOD could be regulated by VEGF and thus might play an important role in brain damage and neuronal cell death (Chen et al., 2019b; Feng et al., 2019; Zhang et al., 2021). The previous study demonstrated that caspase-3 was a key indicative in the process of increased cell death, and the neuronal death due to oxidative stress might present an increase in caspase-3 activation (Jing et al., 2020). In the present study, the expression of NF-ĸB and VEGF in the MCAO/R model group was obviously increased, and an increasing tread but no statistical significance of the caspase-3 level was observed in the MCAO/R model rats as compared with SHAM rats. After treatment with BMD, all of these had a certain improvement. Taken together, changes in oxidative stress played a crucial role in neuroprotective effects against IS injury.

Currently, researchers are increasingly relying on metabolomics to understand the etiology and pathogenesis of IS (Johnson et al., 2016). In this case, UHPLC-QTOF-MS/MS-based metabolomics was carried out to evaluate the protective effects and the possible mechanisms of BMD on MCAO/R rats by employing serum and brain metabolite profiling. A total of 22 differential metabolites in serum and 31 in brain samples between SHAM and MCAO/R groups were identified, and 30 of which were reversed after BMD intervention. These 30 metabolites were mainly related to seven metabolisms, including phenylalanine metabolism, histidine metabolism, tryptophan metabolism, arginine and proline metabolism, glycerophospholipid metabolism, fatty acid biosynthesis, and aminoacyl-tRNA biosynthesis. Amino acids played an important role in IS, and it was found to be involved in many important regulation metabolic processes in organisms (Li et al., 2019). Phenylalanine metabolism, histidine metabolism, tryptophan metabolism, and arginine and proline metabolism are four primary metabolic ways that belong to amino acid metabolism. In this study, the levels of phenylalanine, histidine, tryptophan, valine, and proline were significantly increased in rats of the MCAO/R group as compared to the SHAM rats, suggesting that perturbed amino acid metabolism was implicated in IS. Phenylalanine played a key role in the biosynthesis of cells and tissues, and its hydroxylase could catalyze phenylalanine to tyrosine and further degrade to metabolic products to participate in the tricarboxylic acid cycle (Flydal and Martinez, 2013). More importantly, it could be used for the diagnosis of IS which might further accelerate brain injury and the course of disease (Goulart et al., 2019). Histidine, a precursor of histamine, was an amino acid with antioxidant activity, which was significantly effective in protecting nervous system injury (Tang et al., 2007; Bae and Majid, 2013). Tryptophan metabolism was recognized as a key regulator in the immune system, and an increased kynurenine/tryptophan ratio was proved to be related to neurocognitive function (Ketelhuth, 2019; Hajsl et al., 2020). Previous publications reported that arginine and proline metabolisms took part in many inflammatory diseases, which are integrally linked to cellular metabolism (Xie et al., 2021). Taken together, amino acid metabolism was closely associated with IS, and in our present study, the expression of 20 amino acids in rats of the MCAO/R model group was imbalanced as compared with that in SHAM rats, while BMD could apparently reverse these abnormal metabolites, suggesting that the metabolic disorder *in vivo* could be regulated by the early intervention of BMD.

As the structural components of biological membranes, phospholipids are mainly composed of glycerophospholipids and sphingolipids, which participated in signal transduction (Farooqui et al., 2007). Glycerophospholipid metabolism involved in a lot of living processes, and glycerophospholipid could be classified into six categories, such as PC, PE, and PI according to its different biological functions. Disturbed glycerophospholipid metabolism generally resulted in the rapid production and accumulation of free fatty acid and lysophospholipid, and the IS injury caused by excitotoxicity and neuroinflammation was often accompanied by changes in the lysophospholipid level (Choi and Chun, 2013). In this study, metabolites such as lysoPC (14:0/0:0), lysoPC (20:5 (5Z,8Z,11Z,14Z,17Z)/0:0), lysoPC (18:1 (11Z)/0:0), lysoPC (15:0/0:0), lysoPE (18:0/0:0), lysoPI (18:0/0:0), and lysoPC (16:1 (9Z)/0:0) were all significantly decreased in the MCAO/R group as compared to the SHAM rats. It was worth noting that the changes in the aforementioned metabolites in the MCAO/R rats were all significantly improved after intervened with BMD. All of the results emphasized the importance of amino acid metabolism and glycerophospholipid metabolism in IS and also implied the beneficial effects of BMD on MCAO/R rats.

Having said all of the aforementioned factors, the significant changes in the oxidative stress index, such as NO, SOD, MDA, GSH, and GSSG, indicated excessive ROS production and abnormal oxidative status in MCAO/R rats. ROS was thought to be one of the triggering events for membrane phospholipid degradation in cellular membranes, which was associated with the accumulation of glycerol (Hillered et al., 1998). ROS could also alter protein metabolism or catabolism, thus leading to their degradation into amino acids. As a result, significant changes in the levels of amino acids in MCAO/R rats were observed (Batch et al., 2014). Therefore, the expression of amino acid metabolism and glycerophospholipid metabolism was influenced by oxidative stress to some extent.

## 5 Conclusion

In summary, the present study was the first time to explore the protective effects of BMD on the MCAO/R rat model of IS and the possible mechanisms by the integration of pharmacodynamics and metabolomics. Our results demonstrated that BMD intervention attenuated MCAO/R-induced IS symptoms and brain injuries, as well as improved oxidative stresses. Serum and brain metabolomic profiling suggested that the regulation of amino acid metabolism and glycerophospholipid metabolism had greatly contributed to the neuroprotective effects of BMD. Taken together, BMD might be a potential candidate for IS prevention and treatment.

## Data Availability

The original contributions presented in the study are included in the article/Supplementary Material. Further inquiries can be directed to the corresponding authors.

## Supplementary material

Supplementary Figure S1 Representative TICs of the serum sample in the positive ion mode.

Supplementary Figure S2 Representative TICs of the serum sample in the negative ion mode.

Supplementary Table S1 Method verification of UHPLC-QTOF-MS/MS.

## Abbreviations

BMD, Bai-Mi-Decoction; CCA, common carotid artery; CMC-Na, carboxymethyl cellulose sodium; ECA, external carotid artery; GC-MS, gas chromatography–mass spectrometry; GSH, glutathione; GSSG, glutathiol; HE, hematoxylin and eosin; ICA, internal carotid artery; IS, ischemic stroke; LC-MS, liquid chromatography–mass spectrometry; MCA, middle cerebral artery; MCAO, middle cerebral artery occlusion; MCAO/R, middle cerebral artery occlusion–reperfusion injury model group; MDA, malondialdehyde; NF-κB, nuclear factor-κB; NMDP, nimodipine; NMR, nuclear magnetic resonance; NS, normal saline; NO, nitric oxide; OPLS-DA, orthogonal projections to latent structure discriminant analysis; PCA, principal component analysis; ROS, reactive oxygen species; SOD, super oxide dismutase; TCM, traditional Chinese medicine; TTC, triphenyltetrazolium chloride; UHPLC-MS, ultra-high performance liquid chromatography/mass spectrometry; VEGF, vascular endothelial growth factor; VIP, variable importance in the projection.
