# Supplementary figures and images for: Serum and brain metabolomic study reveals the protective effects of Bai-Mi-Decoction on rats with ischemic stroke
Source: Front Pharmacol. 2022 Nov 24;13:1005301. doi: 10.3389/fphar.2022.1005301 (PMC9729534; doi:10.3389/fphar.2022.1005301)

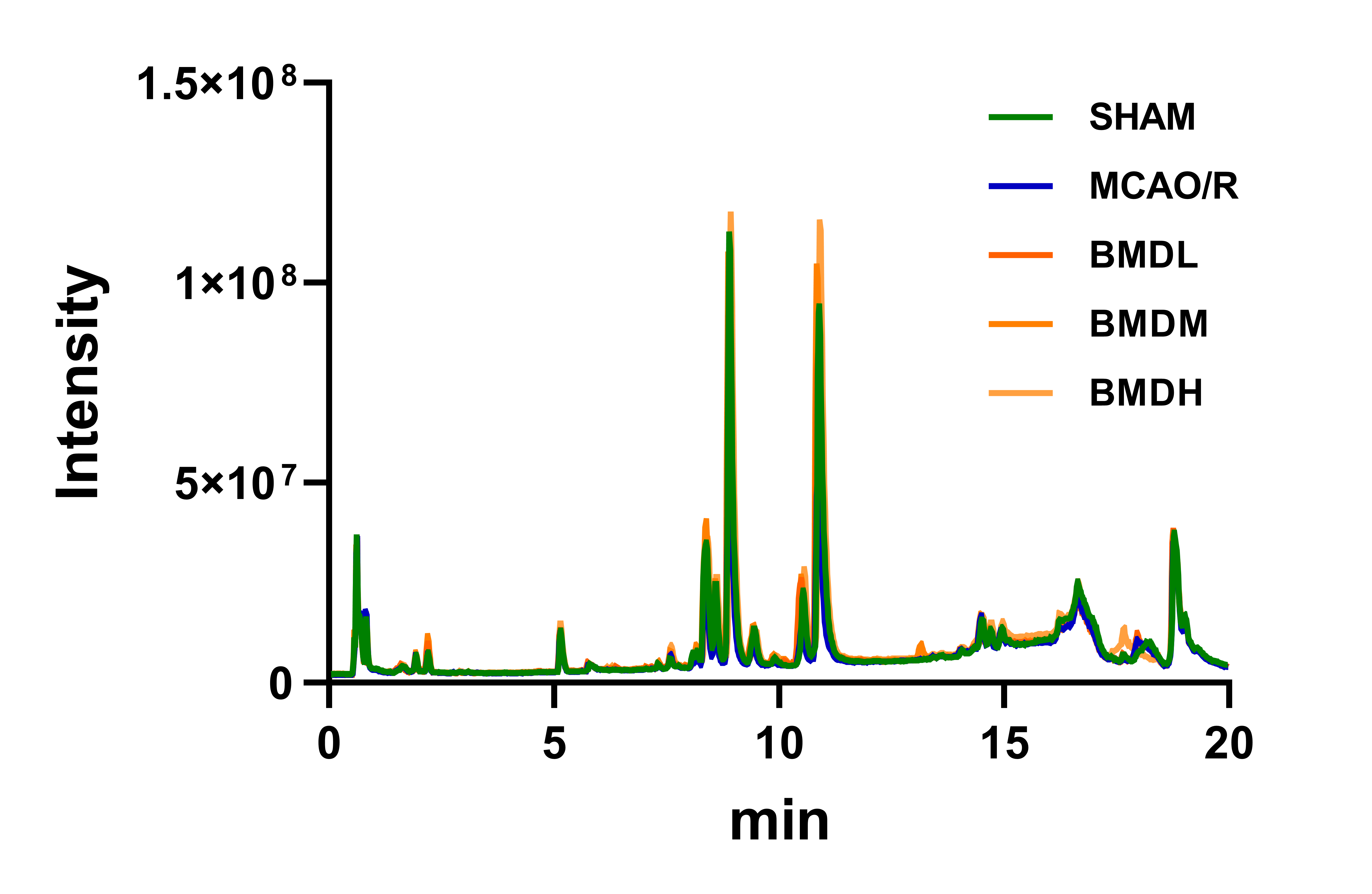


**Supplementary Figure S1** Representative TICs of serum sample in positive ion mode

Supplement: Supplementary file 1 [file Table1.DOCX]

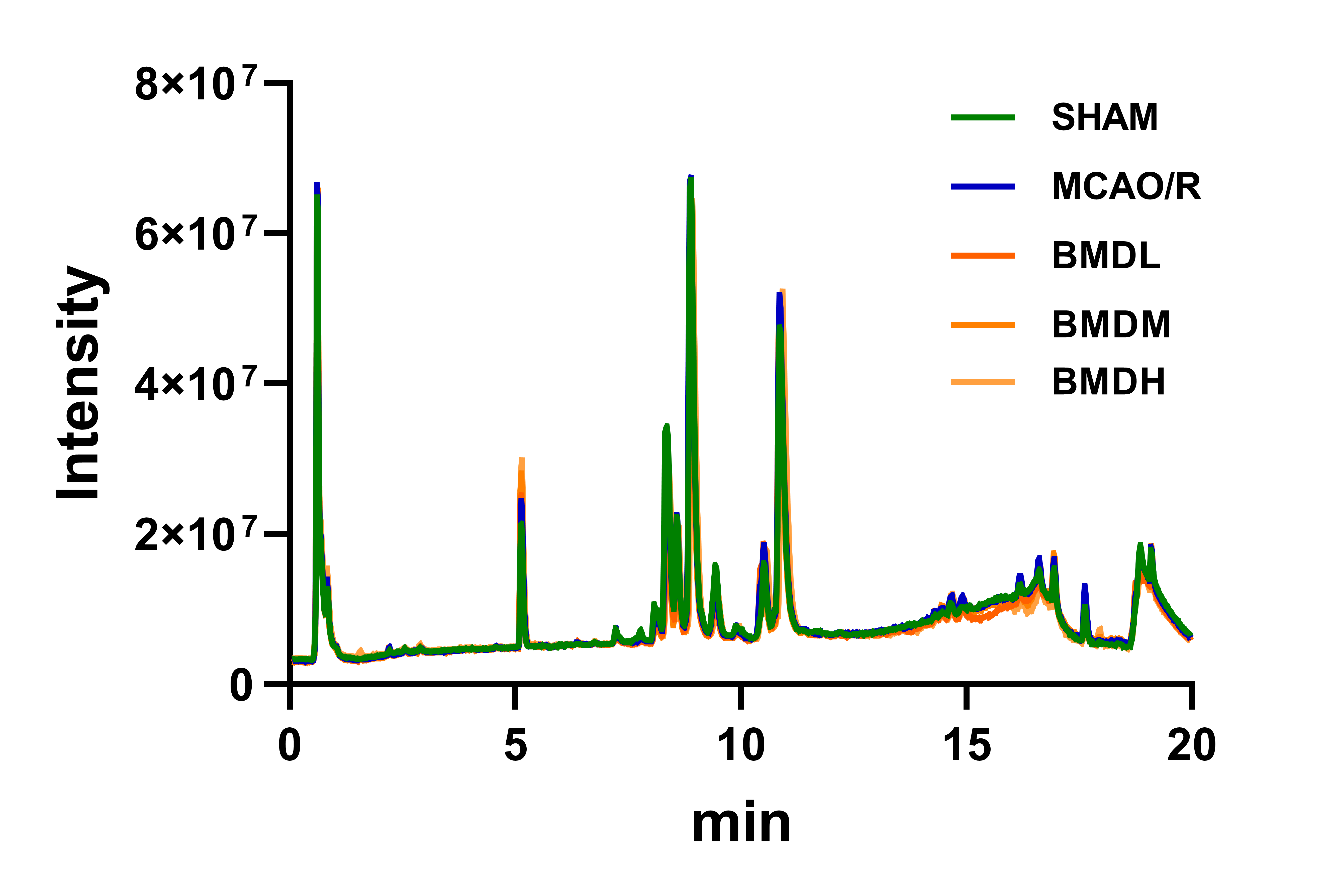


**Supplementary Figure S1** Representative TICs of serum sample in negative ion mode

Supplement: Supplementary file 2 [file Table2.DOCX]
