# Supplementary material for: Serum and brain metabolomic study reveals the protective effects of Bai-Mi-Decoction on rats with ischemic stroke
Source: Front Pharmacol. 2022 Nov 24;13:1005301. doi: 10.3389/fphar.2022.1005301 (PMC9729534; doi:10.3389/fphar.2022.1005301)
Supplement: Supplementary file 3 [file Table3.DOCX]

**Supplementary Table S1** Method verification of UHPLC-QTOF-MS/MS

| sample | [M+H]^+^ m/z | Positive ion mode | | [M-H]^-^ m/z | Negative ion mode | |
| --- | --- | --- | --- | --- | --- | --- |
|  |  | RT *RSD* (%) | Peak area *RSD* (%) |  | RT *RSD* (%) | Peak area *RSD* (%) |
| serum | 188.0715 | 0.05 | 5.13 | 588.3421 | 0.10 | 9.04 |
|  | 415.2125 | 0.29 | 4.78 | 540.3320 | 0.01 | 3.05 |
|  | 520.3406 | 0.11 | 2.55 | 568.3627 | 0.07 | 1.78 |
|  | 496.3410 | 0.06 | 1.03 | 303.2338 | 0.01 | 6.15 |
|  | 524.3719 | 0.07 | 2.49 | 132.9237 | 0.02 | 13.22 |
| brain | 415.2116 | 0.01 | 6.46 | 524.2789 | 0.01 | 1.20 |
|  | 496.3395 | 0.10 | 0.76 | 540.3313 | 0.08 | 3.74 |
|  | 524.3708 | 0.16 | 7.07 | 599.3215 | 0.08 | 6.29 |
|  | 329.2473 | 0.06 | 8.84 | 327.2332 | 0.04 | 6.61 |
|  | 305.2476 | 0.05 | 2.60 | 303.2333 | 0.06 | 6.12 |

RT: retention time
